# Ambiguity in Ethical Standards: Global Versus Local Science in Explaining Academic Plagiarism

**DOI:** 10.1007/s11948-024-00464-6

**Published:** 2024-02-12

**Authors:** Katerina S. Guba, Angelika O. Tsivinskaya

**Affiliations:** Center for Institutional Analysis of Science and Education, European University at St. Petersburg, 6/1A Gagarinskaya St., St. Petersburg, Russia 191187

**Keywords:** Academic plagiarism, Academic misconduct, Academic culture

## Abstract

The past decade has seen extensive research carried out on the systematic causes of research misconduct. Simultaneously, less attention has been paid to the variation in academic misconduct between research fields, as most empirical studies focus on one particular discipline. We propose that academic discipline is one of several systematic factors that might contribute to academic misbehavior. Drawing on a neo-institutional approach, we argue that in the developing countries, the norm of textual originality has not drawn equal support across different research fields depending on its level of internationalization. Using plagiarism detection software, we analyzed 2,405 doctoral dissertations randomly selected from all dissertations defended in Russia between 2007 and 2015. We measured the globalization of each academic discipline by calculating the share of publications indexed in the global citation database in relation to overall output. Our results showed that, with an average share of detected borrowings of over 19%, the incidence of plagiarism in Russia is remarkably higher than in Western countries. Overall, disciplines closely follow the pattern of higher globalization associated with a lower percentage of borrowed text. We also found that plagiarism is less prevalent at research-oriented institutions supporting global ethical standards. Our findings suggest that it might be misleading to measure the prevalence of academic misconduct in developing countries without paying attention to variations at the disciplinary level.

## Introduction

Recently, there has been renewed interest in academic misconduct as a result of switching attention from clearly forbidden forms (e.g., fabrication, falsification, and plagiarism) to questionable research practices (Biagioli et al., 2019). Researchers have documented how universities manipulate figures to improve their positions in international rankings (Espeland & Sauder, 2007), publishers launch predatory journals ready to publish virtually all submissions (Bagues et al., 2019), and individuals resort to gaming strategies like text-recycling (Horbach & Halffman, 2019) and self-citations (Seeber et al., 2019) to enhance their chances for promotion. Research on less severe forms of gaming has produced a shift from descriptive studies conducted by social scientists concerned with such misbehavior in their communities (Biagioli et al., 2019) to the development of explanatory frameworks. As such, studies reveal the fruitfulness of employing different theoretical perspectives from organizational science (Berggren & Karabag, 2019; Hall & Martin, 2019; Walsh et al., 2019), game theory (Gall & Maniadis, 2019), and sociology (Hussinger & Pellens, 2019) to understand academic misconduct. These new theoretical accomplishments provide us with tools to refocus on studying severe forms of academic misconduct, including, particularly, plagiarism.@@@

Why do academics engage in misconduct? Although extensive research has been carried out on systematic causes (Bonn & Pinxtern, 2019), less attention has been paid to the variation in academic misconduct between research fields as most empirical studies focus on one particular discipline. Hence, relatively little is known about how the rate of plagiarism varies across different disciplines, especially in non-Western academia. We propose that discipline is only one of several systematic factors that might contribute to the presence of academic misbehavior. Because few writers have empirically presented the variation in academic misconduct between disciplines, the role of disciplines has not been considered. We suggest a neo-institutional approach to conceptualize the differences in academic plagiarism at the disciplinary level.

From a theoretical standpoint, we follow Honig and Bedi (2012), who have suggested that neo-institutional theory may support a hypothesis that predicts where and when the norm of textual originality may be less espoused by academics. Recent developments in neo-institutional theory show that global pressure to choose legitimate standards results only in a partial convergence due to strong local traditions and institutional memory (Dobbins & Knill, 2009; Hüther & Krücken, 2016; Paradeise & Thoenig, 2013; Stage, 2020). In relation to scientific ethos, we suggest that in the developing countries, the norm of textual originality has not been supported equally across different research fields. Plagiarism especially would be evident in less globalized disciplines since researchers in these disciplines might be less socialized to Western scientific norms and have experienced fewer enforcement actions compared with more globalized academic fields. Our focus on plagiarism in doctoral dissertations provides the opportunity to answer the following question: What level of plagiarism can be observed in texts by Russian scholars that will not be peer-reviewed externally in countries where the norm of originality is prized most highly? Using Russian plagiarism detection software (Antiplagiat), we constructed representative sample of 2405 doctoral dissertations to study the role of academic disciplines in the prediction of research plagiarism. According to Russian law, higher education organizations, research institutes, and academic journals are obliged to use the software to check student papers, submitted manuscripts, and dissertations, making Antiplagiat a widely-used platform.

This study contributes to the existing literature on plagiarism that addresses the systemic factors of academic misconduct. While most research is conducted on Western countries, such as the US, UK, Australia, and Canada (Bonn & Pinxtern, 2019), we collected data from the developing country that allows us to demonstrate the relative character of scientific ethos. Specifically, we hypothesize that non-Western academics might have other views on the seriousness of academic misconduct. Previous studies of the academically less developed countries have revealed that the level of plagiarism in academia is higher when compared with Western countries (Hodges et al., 2017; Lewellyn et al., 2017; Thomas & Bruin, 2015; Vrana, 2018; Xie et al., 2021). These findings confirm Burton Clark’s (1983) statement that an attachment to a global standard without borders is less evident in non-European and non-North American contexts. However, the focus on nation hides the variations that might exist at the disciplinary level as they differ in their attachment to global standards, norms, and practices. In this paper, we not only examine plagiarism across a range of different disciplines but also conceptualize the disciplinary differences in the rate of academic plagiarism depending on the level of its internationalization.

From an empirical and methodological perspective, our contribution is the representative study of academic plagiarism in Russia with the use of undirected methods in data collection. In fact, much of what has been written about plagiarism is based on direct methods when respondents are asked whether they have known colleagues who engaged in plagiarism, or editors could be asked whether they have uncovered plagiarism among submitted papers (Pupovac & Fanelli, 2015). However, the sensitivity of the topic is a critical limitation in self-reported evidence regarding misconduct (Biagioli et al., 2019). As an alternative, measuring unethical behavior by analyzing publications has the potential to overcome this limitation. The employed data collection strategy is an essential contribution because empirical research based on the direct detection of plagiarism in the entire population of scientists in a given country is rare (Bonn & Pinxtern, 2019). In this regard, we expand the previous studies of research plagiarism conducted by the activist association Dissernet (Rostovtsev, 2017); researchers analysed a large sample of Russian dissertations, however, the sample was not representing all disciplines over the entire country, and the attention was focused only on the large-scale plagiarism.

## Global Versus Local Science in Explaining Plagiarism

Research on the causes of academic misconduct includes individual and systemic explanations that more recently have emphasized national and organizational variations. On the country level, the incidence of plagiarism is higher in the developing countries (Hodges et al., 2017; Lewellyn et al., 2017; Xie et al., 2021). In his 2017 survey, Vrana (2018) demonstrated that plagiarized submissions were the second most significant problem experienced by Croatian editors. Xie et al. (2021) conducted a meta-analysis of the prevalence of academic misconduct and found that the rate of self-reported research misconduct is higher in developing countries than in the US and European countries. These empirical findings raise the following question: Why do developing countries have the highest rate of plagiarism?

The literature provides at least several possible explanations. First, there is a suggestion that there exist cultural differences regarding understanding of the notion of plagiarism (Yi et al., 2020). For example, “memorization and repetition have been very important in Chinese education and examinations, and students were encouraged to memorize and repeat literally the words of classic Confucian texts and other reputed historical persons, whereas proper citation (according to Western standards of referencing) was not emphasized” (Yi et al., 2020, p. 2). Although academics from different cultures do not diverge in understanding what constitutes obvious plagiarism (Gupta et al., 2021; Yi et al., 2022; Zhang & Zhang, 2016), there is a difference in understanding of what constitutes plagiarism in practice. Second, the prevalence of plagiarism might be related to the competition between Global North and South that pushes non-native English authors to borrow portions of other’s text to make manuscripts look more professionally written (Biagioli, 2012). While the first explanation emphasizes that different cultural experience prevents authors from fully absorbing scholarly conventions regarding proper referencing and paraphrasing, making academics engage in unintentional plagiarism (EI Bairi et al., 2022), the second explanation refers to the greater rate of tolerance to plagiarism in developing countries.

Both aspects are an issue for developing countries, given that these countries lack ethical legal documents and educational activities or such infrastructures are in the earlier stage of development. In contrast, highly developed countries have elaborated national and institutional frameworks and routinely have educated scientists about ethical behavior through workshops and courses (Pupovac et al., 2017). Even more important is that Western authors consider peer-reviewed scholarship the predominant publication standard (Honig & Bedi, 2012) that facilitates the enforcement of scientific norms. As enforcers of ethical standards, reviewers and editors are expected to provide an editorial process capable of revealing blatant forms of misconduct, including plagiarism (Horbach & Halfman, 2019). In developing countries, research fields differ in the level of absorbing Western experience given that some disciplines are local and their authors rarely engage with Western academic practices—they rarely receive educational experience in US and European universities or rarely have published in international journals, which usually provide the elaborated guidelines regarding academic misconduct.

In our main line of argument, we apply neo-institutional theory (DiMaggio & Powell, 1983; Meyer & Rowan, 1977) to the academic disciplines. The neo-institutional theory focuses on socially constructed beliefs, norms and rules that shape individual and organizational actions. The theory highlights that norms are not universal; rather they are situated in specific social and historical contexts (Shadnam & Lawrence, 2011). This approach allows considering academic disciplines as a nested field, meaning that disciplines, as well as organizations, are embedded in the national field and others, including global fields, which are the source of legitimate models (Hüther & Krücken, 2016). Regarding the academic field, Drori and Moon (2006) cited the collapse of the Soviet bloc as an example of the reorientation of science from the dominance of communist ideology toward the Western core. At the same time, the empirical evidence suggests that contrary to the convergence thesis, global models are pragmatically transformed—resulting in dissimilar practices (Hüther & Krücken, 2016; Paradeise & Thoenig, 2013; Stage, 2020). Drori and Moon (2006) pointed out that, due to country differences in the intensity of scientific work, the level of government commitment and available resources might contribute to divergent tendencies from the global trend. Honig and Bedi (2012) studied management as a discipline developed in North America and only later spread to other countries and found a link between non-core countries and plagiarism, confirming that norms and practices may only be partially reproduced in developing countries.

The concept of nested fields lets us understand how global academic standards, including ethical ones, are reproduced in the context of developing countries. Although Western academia is a significant source of influence; however, it is not uniform for all research fields depending on the level of its internationalization. Authors from more globalized disciplines face Western standards more often as they publish internationally, making them conform to standards that reviewers enforce. However, not all disciplines are internationalized and the local embeddedness might prevent them from fully absorbing a Western standard. Some countries fully absorb the necessity to publish in international outlets (Hokka, 2018) while other communities have divided attitudes towards globalization (Gantman & Fernandez, 2016; López & Hicks, 2015). Post-Soviet sociology is the example of a discipline divided between “those who identify themselves with international and global science with those who are oriented towards predominantly local debates and audiences” (Sokolov, 2018, p. 5). Following the collapse of the Soviet Union, Western foundations supported the creation of a “global” sociology—they distributed research grants and scholarships, gave money for academic travel, and provided institutional support for Western-style private research centers and universities that espoused an opposite mission than the older public institutions (Sokolov, 2018). However, academic groups oriented toward local traditions are developed in parallel to those based upon “Western” values.

Given the relational character of academic misconduct Biagioli (2012), we propose that the necessity of following the Western ethical standard would be different for those who engage in the international community and those who prefer to stay local and avoid publishing internationally. Local groups might implement their own norms and practices as they are able to maintain their resource base. Thus, we propose our main hypothesis.

**H1**: *The incidence of plagiarism will be higher in less internationalized disciplines than those from more internationalized scientific fields*.

While the main focus of the paper is variance at the disciplinary level, we recognize that other structural factors might be relevant. We further suggest including organization-oriented factors to bring attention to the idea that misconduct emerges as a result of organizational or institutional incentives (Greve et al., 2010). Following Greve et al. (2010), we propose that less plagiarism is expected at institutions that possess a concentration of the top researchers in their field. First, those institutions enjoy stronger faculty members and likely more effective oversight, and they have more to lose (Greve et al., 2010, p. 64). On the other hand, research institutions support “publish-or-perish” pressure that might stimulate authors to cut corners to satisfy increased requirements (Davis et al., 2007; Fanelli et al., 2018). The latter hypothesis was tested empirically, demonstrating that only blatant cash incentives influence the risk of scientific misconduct (Fanelli et al., 2018). Thus, we hypothesize that universities engaged in global competition should demonstrate a convergence with the model of world-class universities, including supporting global standards in their academic ethos. Based on this line of research, we propose:

***H2***: *The incidence of plagiarism will be less evident for scholars whose defense was held at research-oriented organizations.*

## Data and Methods

### Antiplagiat Software

We used Russian plagiarism detection software (Antiplagiat), a tool allowing for the comparison of selected texts with extensive text collections, particularly dissertations and academic publications available through the integration with Russian Scientific Library and Russian Scientific Electronic Library (eLIBRARY.RU). The Russian State Library contains a comprehensive digital collection of more than 919,000 full texts of Russian dissertations (Kopotev et al., 2021). eLIBRARY.RU module incorporates many categories of scientific publications, including journal articles, books, and book chapters. The main feature of eLIBRARY.RU is comprehensive coverage because the electronic library does not have strict policy for selecting scholarly periodicals (Moskaleva et al., 2018). To date, the journal catalog of eLIBRARY.RU comprises 14,551 active periodicals with 13,761 open-access sources. The total number of indexed publications is 41 million, and 15 million full texts are available at eLIBRARY.RU. Recently, Antiplagiat was used to study academic ethics violations in Russian scientific periodicals related to the duplicated publication (Chekhovich & Khazov, 2022). Thus, Antiplagiat is a reasonable instrument for detecting academic plagiarism.

Antiplagiat provides a report for each document search that outlines problematic and non-problematic text matches; the latter includes identified self-citations and citations.^1^ Like similar software, Antiplagiat gives scores for each problematic match, defining the total as the share of all text overlaps found by the program except for those that the system classifies as citations and self-citations. Given that Antiplagiat is merely text-matching software, users are advised to consult experts before deciding whether a text has been plagiarized, as the text-similarity score provides information but not a conclusion.

### Sample

We focus on plagiarism in doctoral dissertations that relatively experienced scholars produce, as a doctoral dissertation in the Russian context is a higher degree that may be awarded after receiving the Candidate of Sciences. Doctor of Science is a degree that is comparable to the degree of doctor habilitates in some European countries. The degree requires authors to contribute significantly to a relevant discipline that should be confirmed by publishing at least ten journal articles in natural sciences and 15 articles in social sciences and humanities in addition to the dissertation manuscript. Our focus on doctoral dissertations, not on academic journal publications or books, allows us to avoid the limitation related to differences in disciplinary publication practices. An examination of journal papers, for example, would leave out other relevant publication formats; thus, dissertations help us to study plagiarism across different disciplines.

Our data were constructed in the following steps. First, we retrieved the list of all doctorate degrees (27,735) awarded in Russia between 2007 and 2015 from the official site of the national state agency responsible for overseeing academic degrees. Then we randomly selected 2600 doctoral dissertations from the pool of all dissertations defended to be searched for available text. We found 2468 texts of dissertations, which is 8.9% of all dissertations defended at that time. For further analysis, we excluded a small number of dissertations that were awarded in cultural studies and art history as their number was too low for any reliable analysis. The final sample contains 2405 dissertations written in the Russian language.^2^ Our approach aimed to develop a representative sample of dissertations that we consider as significant contribution given that other researchers (first of all, a volunteer network called Dissernet), although checked several hundred thousand dissertations, used non-representative samples to study plagiarism in Russian dissertations (Rostovtsev, 2017).

### Variables

Ideally, plagiarism should be identified by a human expert who reviews the textual similarity results and make conclusion based on the context. Human inspection is required to detect actual plagiarism; however, relying on experts might be time-consuming in the case of large samples. Most studies that estimate plagiarism based on text-matching software and additional human verification have analyzed a substantially small number of papers, fewer than 1000 (Pupovac, 2021). One possible way to deal with false positives is to use a percentage similarity cut‐off as a threshold, a process broadly implemented by academic journals that receive a significant number of submissions (Manley, 2021). As such, a high value of textual similarity, i.e., more than 50% of textual overlap, helps to eliminate false positives (Pupovac, 2021). However, there is no consensus on the acceptable extent of text similarity in scholarly articles (Gupta et al., 2021) and this method detects only the most obvious cases of text-based plagiarism. Besides, even low percentage of similarity can indicate plagiarism if a borrowed text was reproduced from one source without proper reference.

The specifics of Antiplagiat allow to consider text overlaps that do not have a proper reference as plagiarized texts given that Antiplagiat is capable, to some extent, automatically eliminate false positives, e.g., matches that are not actually plagiarism. First, it identifies text in quotes that have been cited correctly. The software defines a quotation as text in quotation marks that satisfies at least one of the conditions: (1) before the quote, the name of the author and a word denoting an utterance (e.g., speaks, writes, asserts) are present; (2) after the quotation, there is a link to the reference in square brackets or parentheses, or there is a footnote after the quote. It is critical that Antiplagiat can handle different citation styles, including cases of locating references in the footnotes allowing to consider text overlap as cases without the use of quotation marks and a precise reference to the original source. Second, Antiplagiat reviews non-problematic matches like template phrases (e.g., the names of universities and authoritative bodies, introductory words, stable speech turns, judicial acts) and matches them with collections of normative documents. Third, the software automatically identifies a bibliography (list of references), regardless of the formatting standard and language, and excludes it from analysis. Finally, Antiplagiat provides the opportunity to eliminate false positives resulting from text reuse when the software detects matches with texts published earlier by the same author. More important is the possibility of applying the special algorithm, which detects the texts published after the year in which the dissertation was defended as non-problematic.^3^ Thus, this algorithm signals that an author plagiarized in his/her dissertation because the cases when his/her texts were plagiarized are not taken into consideration.

In addition, Antiplagiat’s setting allow an evaluator to exclude minimal matches, which might be non-problematic, by using a percentage threshold. This elimination increases the precision in labeling matched text as plagiarized text. There are different approaches to using thresholds to detect plagiarism through software, from three consecutive words to 30-word strings of text-matching (Hodges et al., 2017; Sun, 2013). For this research, a percentage of 0.01 was used to identify text we consider legitimate. On average, this percentage is equal to eight words (the mean number of words in a dissertation is 78,274). In our decision, we follow suggestions of other researchers. For example, Citron and Ginsparg (1995) stated that a text match of more than seven words might be considered problematic. Hodges et al. (2017) chose a minimum 10-word string of text-matching without attribution. Gupta et al. (2021) suggest from the personal experience, a limit of at least eight to ten words should be used to avoid detecting unproblematic similarity.

**Fig. 1:**
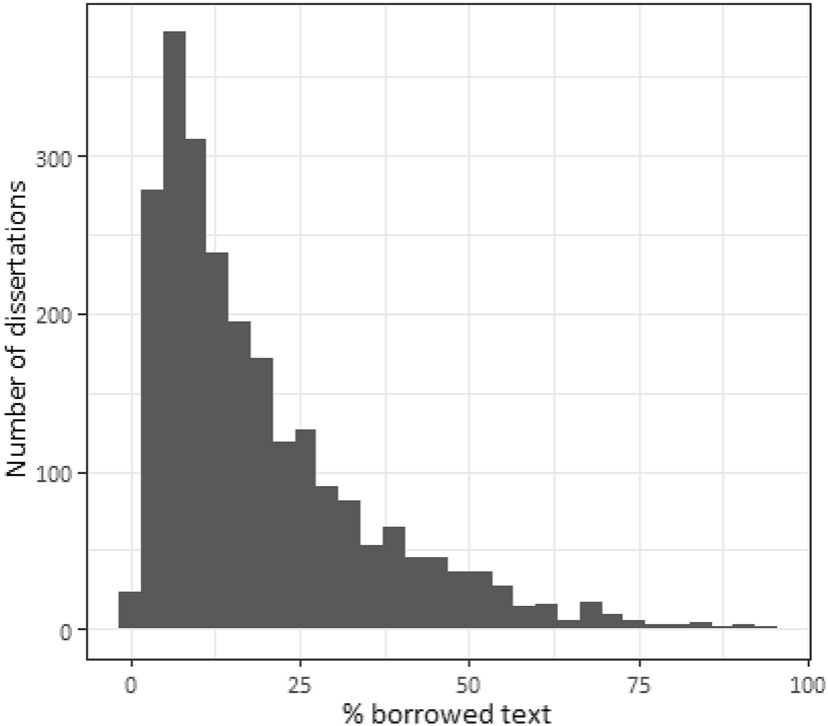
Distribution of borrowed text

Thus, we consider text overlaps that do not have a proper reference as plagiarized texts and use the percentage of borrowed text as the main dependent variable. Figure 1 presents the overall distribution of borrowed text for all dissertations in our final sample. Looking at the overall shape of the distributions (Fig. 1), we conclude that the incorrect borrowing is widespread, but cases of entire copy-paste works are rare.

For the analysis of our hypotheses, we relied on Scopus and the Russian Index of Science Citations (RISC) to collect data on the level of internationalization of academic disciplines. RISC, launched in 2005, is integrated with a full-text platform, the Scientific Electronic Library, which indexes more periodicals than RISC (Moskaleva et al., 2018). Reliance on a national source of bibliometric data is critical given that we study the non-Western higher education system, where the research is not always indexed by Web of Science or Scopus (Mongeon & Paul-Hus, 2016).

From different strategies to measure the globalization of academic disciplines, we chose to calculate the share of publications indexed in the global citation database in the overall output of academic disciplines. To calculate the number of publications produced in the global context, we queried Scopus for articles written in English with Russian affiliation in 2018 that correspond to academic disciplines in Russia (we grouped subject areas when needed and also used sub-subject areas to meet this condition). As for the number of publications produced in the local context, we relied on RISC, which covers the output of Russian journals, grouped by academic disciplines. We queried publications in Russian journals written in Russian in 2018. We limited our search to 2018, as earlier coverage of publications is known to be of less satisfying quality. Globalization was calculated as:

1 $$globalization_{i} = \frac{{n_{i}^{Scopus} }}{{n_{i}^{Scopus} + n_{i}^{RICS} }} \cdot 100\% \quad ,i = \overline{1,m} ,$$

where *m* is the number of disciplines (in our case, we considered 16), $n_{i}^{Scopus}$ is the number of publications in Scopus *i*-th discipline, and $n_{i}^{RICS}$ is the number of publications in RICS *i*-th discipline.

We calculated the value of globalization as highest for physics and math, with 45.5%, and lowest for the legal field, with only 0.2% (Table 1). The ordering of the academic disciplines by globalization can be considered as supporting the divide between the natural and social sciences.

**Table 1:** Globalization by disciplines

Discipline	Globalization (%)	Median for borrowed text (%)
Physics and math	45.54	6.2
Chemistry	29.32	20.68
Biology	13.22	11.96
Earth science	11.48	8.69
Technical	9.75	14.38
Agriculture	4.79	29.12
Medicine	2.71	15.16
Economics	1.82	18.26
Philosophy	1.59	8.6
Psychology	1.11	12.71
History	1.08	10.29
Sociology	0.89	12.24
Political science	0.89	11.93
Education	0.58	15.27
Literature	0.51	6.86
Law	0.21	25.61

In addition, the globalization of academic disciplines was calculated alternatively based only on the Russian national database, RISC, which provides access to each institution profile, including universities and research institutions. Information from the institution profile contains the overall number of publications and the number of publications indexed in the international databases Web of Science (WoS) and Scopus separately. We chose to collect information only for WoS since this citation database is known to have less representation of national journals published in languages other than English^4^ (Mongeon & Paul-Hus, 2016). Data on the number of publications was available only for six research fields: fundamental science, engineering science, medical science, social science, humanities, and agricultural science.

As such, we were able to calculate the level of globalization only for these broad fields. Globalization was calculated as:

2 $$globalization_{i} = \frac{1}{N}\sum\limits_{j = 1}^{N} {\frac{{n_{ij}^{WoS} }}{{n_{ij}^{RICS} }}} \cdot 100\% ,\quad i = \overline{1,m} ,$$

where *m* is the number of fields (in our case, we considered six), $n_{ij}^{WoS}$ is the number of publications in WoS *i*-th discipline by *j*-th organization, and $n_{ij}^{RICS}$ is the number of publications in RISC *i*-th discipline by *j*-th organization. *N* is the number of organizations.

We retrieved data for about 2305 organizations registered in the RISC database with institutional profiles. The calculated value of globalization is the highest for the natural sciences field, with 22.1% (Table 2).

**Table 2:** Globalization by fields

Field	Globalization (%)	Median for borrowed text (%)
Fundamental science	22.1	9.9
Engineering science	6.8	14.4
Medical science	6.4	15.2
Humanities	5.6	8.6
Social science	5.3	17.2
Agricultural science	3.3	29.1

We used log transformation because the percentage of borrowed text has a skewed distribution with the heavy right tail. The results show that the level of globalization calculated both by disciplines and by fields is negatively correlated with the percentage of borrowed text, and it is highly statistically significant (Pearson’s correlation is r =− 0.176***, *p* < 2.2e−16 for globalization by disciplines and r =− 0.159***, *p* = 4.851e−15 for globalization by fields).

Further, we collected information on the type of institution where the defense was held. Less plagiarism is expected at institutions such as the Academy of Sciences and research universities, including participants of the Russian Excellence Program “Project 5–100.” Historically, research has been concentrated at the Academy of Sciences and not in universities. Project 5–100 was launched in 2012 to improve the international competitiveness of a handful of Russian universities. As such, universities were encouraged by financial incentives to implement the model of a world-class research university. Consequently, we coded them as research-oriented organizations and others as not research-oriented. There are 26.9% dissertations in our sample that were defended at research-oriented organizations (Table 3).

**Table 3:** Summary statistics for variables of interest

Variables	Values	Frequency (N)	Percentage (%)	% Borrowed text				
				Min	Mean	Median	Max	Std. deviation
Gender	Male	1331	55.3	0.5	19.32	14.18	91.83	16.8
	Female	1074	44.7	1.39	19.3	14.18	94.38	15.17
Defense year	2007	66	2.7	1.39	20.11	17.16	89.09	15.8
	2008	327	13.6	1.98	19.47	14.28	74.03	15.84
	2009	533	22.2	0.5	20.04	14.03	88.79	17.2
	2010	307	12.8	0.64	20.03	14.93	81.07	16.62
	2011	367	15.3	0.76	19.98	14.35	84.51	17.12
	2012	246	10.2	1.29	20.1	16.03	89.25	15.48
	2013	184	7.6	2.01	19.43	15.61	94.38	15.48
	2014	249	10.3	1.58	17.43	13.47	80.7	13.87
	2015	126	5.2	0.92	13.7	9.59	91.83	12.4
Organization type	Research-oriented	648	26.9	0.5	15.14	10.16	89.09	14.03
	Not research-oriented	1757	73.1	0.69	20.85	16.16	94.38	16.53
Discipline	Agriculture	93	3.9	3.01	31.16	29.12	84.51	18.84
	Biology	121	5.0	1.88	19.23	11.96	80.81	16.46
	Chemistry	67	2.8	1.02	25.81	20.68	81.07	19.14
	Earth science	56	2.3	0.86	11.66	8.69	57.94	11.17
	Economics	435	18.1	2.29	22.99	18.26	85.83	16.75
	History	128	5.3	1.43	14.98	10.29	91.83	14.98
	Law	106	4.4	4.53	27.86	25.61	67.61	13.51
	Medicine	329	13.7	1.98	19.18	15.16	88.79	13.94
	Education	203	8.4	2.54	20.27	15.27	89.25	15.95
	Literature	116	4.8	0.84	9.18	6.86	61.04	8.52
	Philosophy	91	3.8	1.92	12.18	8.6	47.86	10.06
	Physics and math	164	6.8	0.5	12.55	6.2	94.38	15.33
	Political science	40	1.7	3.15	16.06	11.93	45.55	11.02
	Psychology	52	2.2	3.89	17.53	12.71	62.89	14.3
	Sociology	46	1.9	2.23	14.38	12.24	41.51	9.6
	Technical	358	14.9	0.69	19.81	14.38	74.18	17.18
	Total	2405	100					

In addition, the analysis included two other independent variables as controls: gender and the defense year of a dissertation. These variables are included in line with previous research and possible variation that can be attributed to them in the models. Gender is considered to be a possible relevant factor since males might be more inclined to take risks than females, and might therefore be more likely to engage in academic misconduct (Fanelli et al. (2018) proposed the gender hypothesis although the results of the study did not support it). Table 3 presents the gender distribution of doctorate holders in our sample. There are more male authors (55.3%) than female ones. We also included the defense year of a dissertation as, over the years, some reformation of the system has occurred during a ten-year span in regards to the process of obtaining the doctoral degree, making it more difficult to falsify works and raising the quality of defended theses. Defense year varies between 2007 and 2015. This period was chosen due to the availability and sufficient coverage of information on dissertations.

## Results

### Descriptive Statistics

**Fig. 2:**
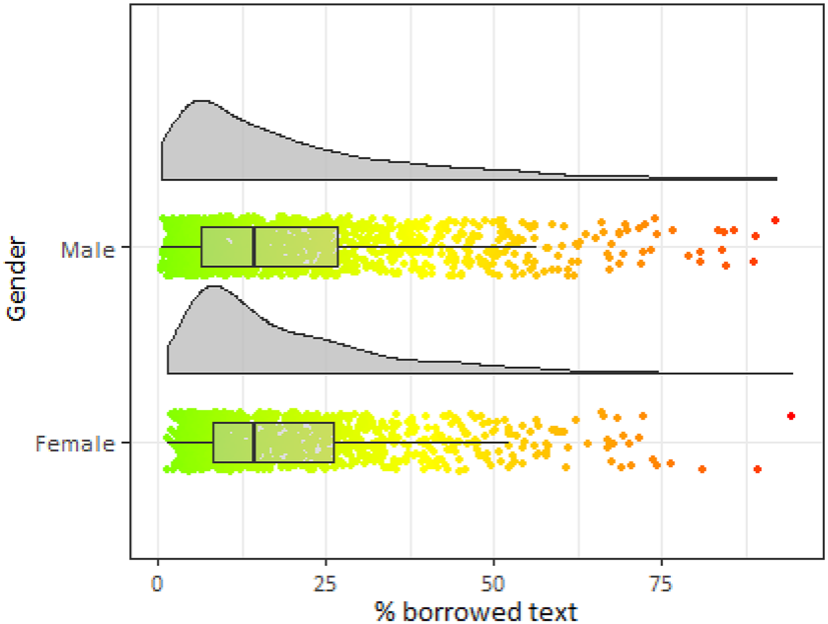
Distribution of borrowed text by gender

**Fig. 3:**
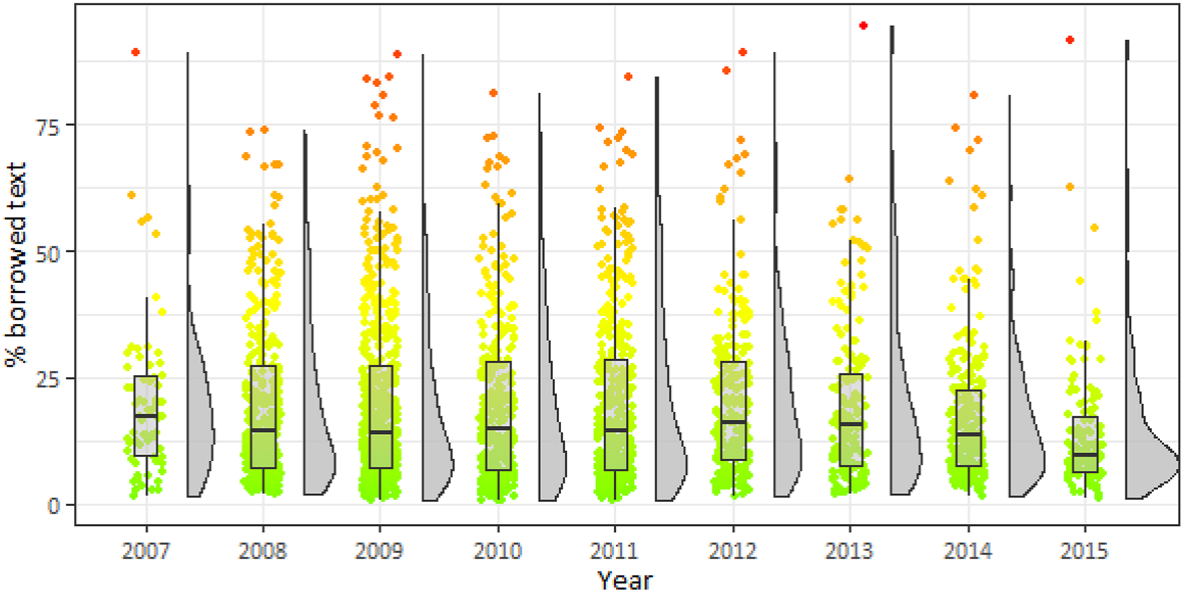
Distribution of borrowed text by year

**Fig. 4:**
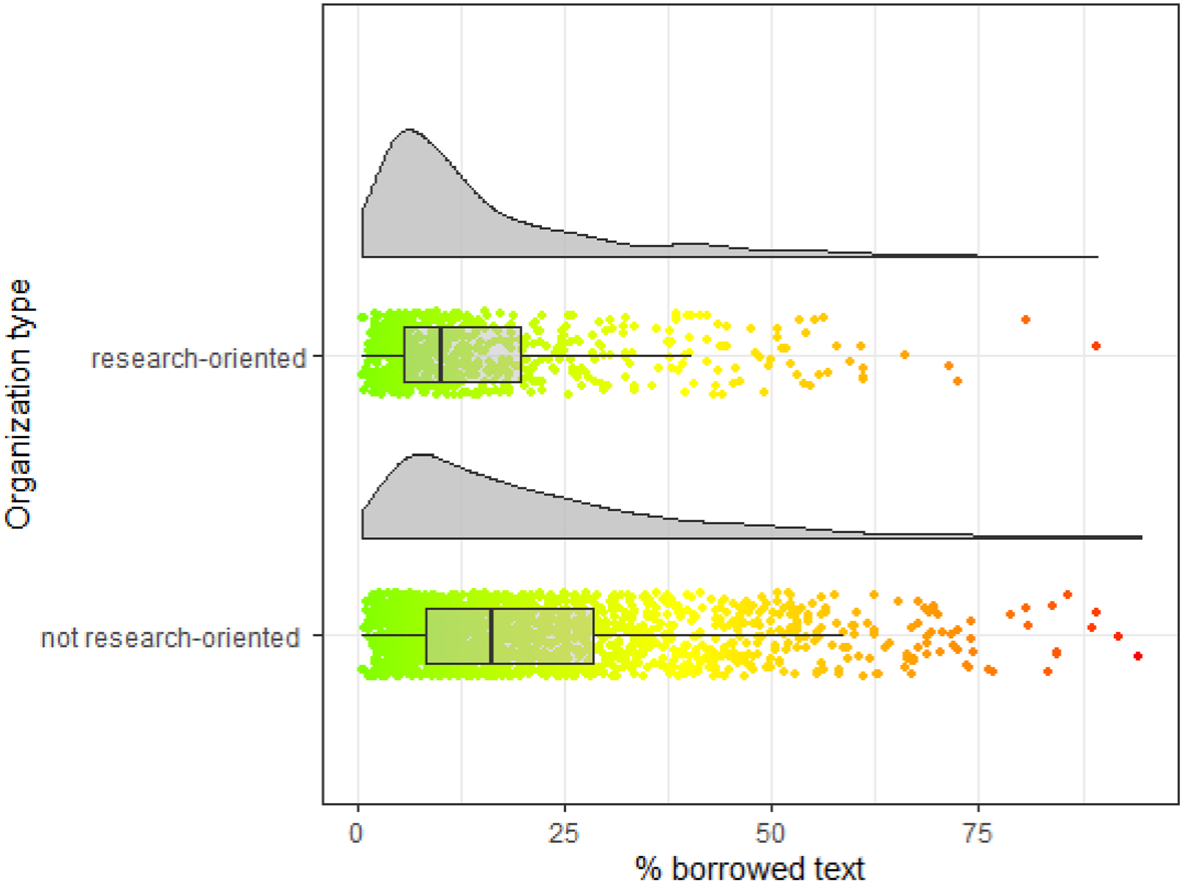
Distribution of borrowed text by organization type

**Fig. 5:**
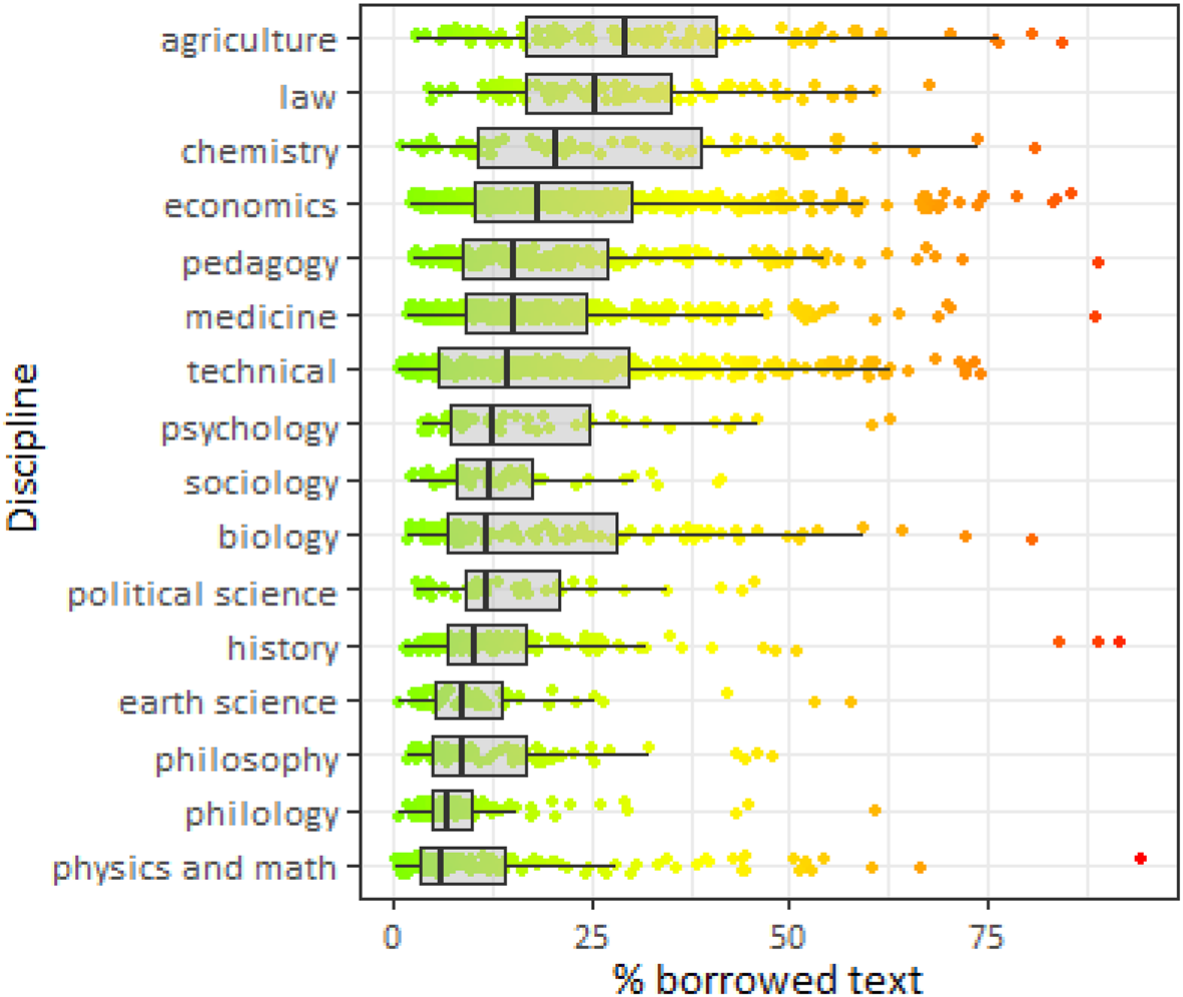
Distribution of borrowed text by discipline, ordered by median percentage of borrowed text

As a starting point for our analysis, we turn to the graphical approach to show its key features and better depict the nature of our variables. Using this data visualization approach, we show differences in the percentage of borrowed text for selected variables such as gender, the year of defense, the organization type, and discipline (Figs. 2, 3, 4, and 5).

Our data demonstrate that the average share of detected borrowings was 19.1% (median: 13.9%). Only a quarter of dissertations have a level of plagiarized text less than 7%. At the same time, it is rather unusual to copy the entire text: in the sample of 2468 dissertations, we found only 41 where borrowing exceeded 50%.^5^ Figure 2 illustrates that the distributions of borrowed text for males and females are similar in shape. There are slightly more observations, with more than 75% of borrowed text, for men. Both men and women have a median value of 14.18% with the standard deviation is 16.8% for men and 15.17% for women.

We considered the defense year of the dissertation as another common control variable (Fig. 3). The biggest difference in the percentage of borrowed text is for 2007 compared to other years (the median is 9.59%). The Mann–Kendall Trend Test for median percentage of borrowed text showed (tau = − 0.333, 2-sided *p *value = 0.25) that a trend is not statistically significant.

Regarding the influence of organizational type on the percentage of the detected plagiarized text, we can distinguish between research-oriented and not research-oriented organizations (Fig. 4). The difference in median values is around 6% less borrowed text at research-oriented organizations. The distribution for research-oriented organizations is less spread out and has a higher peak. There is some evidence for the effect on borrowed text by organization type where the dissertation was defended.

Finally, we show how academic discipline affects the distribution of borrowed text (Fig. 5). The highest median percentage of borrowed text is in agricultural dissertations (29.12%), legal sciences (25.6%), and chemical sciences (20.8%). The lowest is in physics and math (6.2%), literature (6.9%), and philosophy (8.4%) that is line with the observation made on the study of text-recycling in four research areas—Horbach and Hoffman (2019) found that humanities are disciplines for which the wording is the essence of the novelty. We found that in the social sciences, the incidence of plagiarism is evident for economics, education, psychology, and political science. Our results generally do not contradict the previous attempts to study plagiarism in Russian dissertations. Thus, Dissernet, an activist association, found large-scale plagiarism in over 6500 dissertations and conclude that plagiarized texts are primarily produced in economics, pedagogy, and law, followed by medical sciences, political sciences, engineering, and social sciences. In contrast, plagiarized dissertations are rare in natural sciences (Rostovtsev, 2017).

**Fig. 6:**
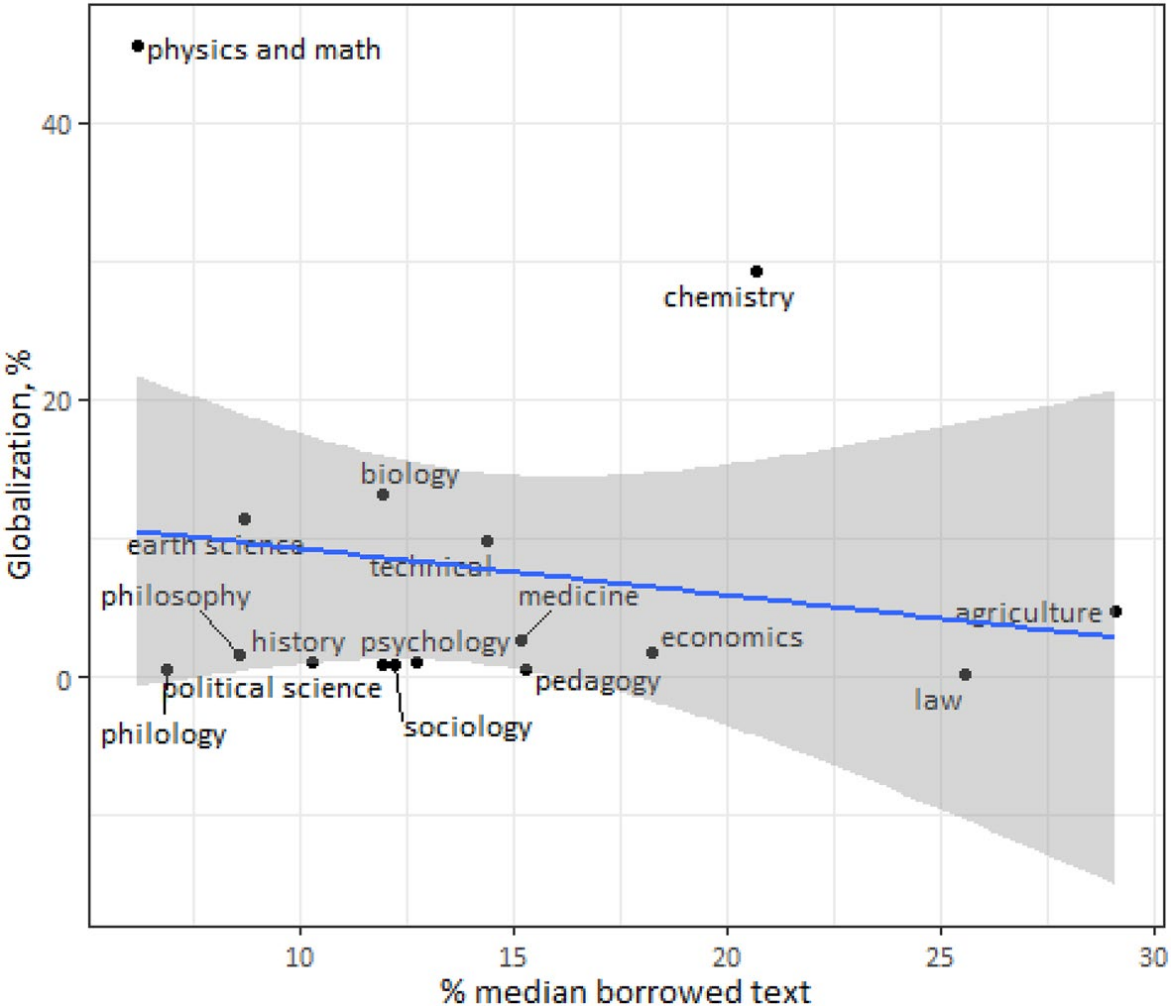
Median % of borrowed text by globalization of discipline

As our primary variable of interest is globalization, we engaged in a closer investigation of the relationship between globalization and the percentage of borrowed text (Fig. 6). Physics and math stand out as having the highest level of globalization and the lowest median value for borrowed text. Dissertations in chemistry have a higher median percentage compared to other natural disciplines, which are more globalized than the social sciences. Other disciplines that more closely follow the pattern of higher globalization are associated with a lower percentage of borrowed text.

### Regression Models

Values of our dependent variable can be expressed as bounded by the interval (0,1). We considered several regression models, which are generally the appropriate choice for proportional data. Our selection suggested that the CDF-quantile model best describes the data, as it out-performed the other ones such as beta and fractional models. This model has additional properties compared to alternatives as it not only distinguishes the effect median difference but also the effect of independent variables on dependent variables in terms of the variability of values, as it has two submodels (location and dispersion). There is a relation between pairs of these distributions. These pairs are “quantile-duals” of one another in the sense that one’s CDF is the other’s quantile, with the appropriate parameterization. Separate submodels may be specified for the location and dispersion parameters, with different or overlapping sets of predictors in each (Smithson et al., 2017).

We present the general equation for the CDF-quantile regression model for random variables on the $\left( {0,1} \right)$ interval (Smithson et al., 2017). Consider a random sample of *N* independent observations from $\left( {Y,{\mathbf{V}}} \right)$, where $Y$ is a random variable and ${\mathbf{V}}$ is a vector of predictors. A model for the distribution of $Y$, conditional on ${\mathbf{V}}$, has two submodels: the “location submodel” and the “dispersion submodel.” The location submodel has a vector ${\mathbf{W}}$ of predictors selected from ${\mathbf{V}}$, and the dispersion model has another vector of predictors ${\mathbf{Z}}$ of predictors selected from ${\mathbf{V}}$. The sets of predictors in ${\mathbf{W}}$ and ${\mathbf{Z}}$ may not overlap. For the *i*-th observation, these submodels may be written as:

3 $$\begin{array}{*{20}c} {L_{\mu } \left( {\hat{\mu }_{i} } \right) = {\mathbf{w}}_{i}^{\text{T}} {{\varvec{\upbeta}}}\;} \\ {L_{\sigma } \left( {\hat{\sigma }_{i} } \right) = {\mathbf{z}}_{i}^{\text{T}} {{\varvec{\updelta}}}} \\ \end{array} ,\;i = \overline{1,N} .$$

For submodels in the CDF-quantile regression, different families of fitted distributions may be chosen. There are no clear criteria for the selection process, but based on the log-likelihood measure, our choice fell on T2-T2 distributions.

The full model includes control variables and both the globalization and the organization type in the location submodel and constant representing the spread of distribution for the response variable in the dispersion submodel (specified in Eq. 4):

4 $$\begin{gathered} \hat{\mu }_{i} = \beta_{0} + \beta_{1} gender_{i} + \beta_{2} defense\_year_{i} + \beta_{3} organization\_type_{i} + \beta_{4} globalization_{i} \hfill \\ \log \left( {\hat{\sigma }_{i} } \right) = \delta_{0} + \delta_{1} globalization_{i} . \hfill \\ \end{gathered}$$

The results of regression analysis with the specified model in Eq. 4 for two calculations of globalization by fields and by disciplines are presented in Table 4. In the descriptive analysis, we presented variables that might influence the percentage of borrowed text separately and in the aggregate form. In contrast, models below contain data on the individual level, taking into account the influence of variables together.

**Table 4:** CDF-quantile regression models

	By fields	By disciplines
**Location submodels**		
Intercept	− 1.556 (0.138)***	− 1.662 (0.133)***
*Control variables*		
2008	− 0.111 (0.135)	− 0.106 (0.137)
2009	− 0.118 (0.13)	− 0.109 (0.131)
2010	− 0.046 (0.136)	− 0.021 (0.138)
2011	− 0.128 (0.135)	− 0.111 (0.136)
2012	− 0.029 (0.137)	− 0.007 (0.138)
2013	− 0.106 (0.142)	− 0.091 (0.143)
2014	− 0.207 (0.136)	− 0.192 (0.138)
2015	− 0.546 (0.152)***	− 0.529 (0.153)***
male	0.064 (0.045)	0.078 (0.046)*
*Variables of interest*		
Globalization	− 1.744 (0.45)***	− 1.039 (0.25)***
Not research-oriented	0.458 (0.056)***	0.467 (0.055)***
**Dispersion submodels**		
Intercept	− 0.209 (0.022)***	− 0.208 (0.022)***
Num. obs	2405	2405
Log likelihood	1649.491	1650.465

Regarding the controls, there is no statistically significant gender difference in the percentage of plagiarized text in the model by fields and a low statistically significant difference in the models by disciplines. Regarding the defense year, the trend test was not statistically significant; the only statistically significant difference in the models is between 2015 and 2007. The median value at the beginning of the period was 17.16%. In the next two years, it lowered to around 14%, and in 2012, it was higher with a median of 16.03%, while at the endpoint of the observed period, it was 9.59%. The beginning and endpoint of the period have a smaller number of dissertations, so we cannot reach a definitive conclusion about this difference and a slowing trend in the percentage plagiarized over the years. Overall, no statistically significant observable trend or pick differences are associated with possible interventions in a particular year.

The results show that with the increase in the level of globalization calculated by disciplines, the percentage of borrowed text lowers. The model shows a negative coefficient in the location submodel (estimated coefficient = − 1.039, *p* < 0.001, which is the median difference. In case of globalization calculated by fields, the higher is the level of globalization, the lower is the percentage of borrowed text (estimated coefficient = − 1.744, *p* < 0.001). In both calculations, the coefficients for globalization in the models are highly statically significant. Thus, our hypothesis H1 about globalization is confirmed based on the regression analysis results. We also found a highly statistically significant difference between research-oriented and not research-oriented organizations, with the median values of 16.16% and 10.16%, respectively. Models show that the median value is lower by 0.467 (0.458) for research-oriented organizations with globalization calculated by disciplines (fields), indicating that the H2 hypothesis is also confirmed. Dissertations associated with research universities and the Russian Academy of Science have, on average, fewer plagiarized texts (compared to median values).

## Discussion and Conclusion

This study was designed to determine the role of academic disciplines in addition to individual and organizational factors relevant for explaining the rate of plagiarism. To study academic plagiarism, we constructed unbiased sample of Russian doctoral dissertations which were analyzed through an online plagiarism detection service. The results suggest that the rate of incorrect borrowing is substantial among Russian degree holders. With the threshold at 20% plagiarized text, more than a third of dissertations (37%) were flagged that is remarkably higher than in Western countries [at this level of threshold, the study showed that 5% of publications from the United States and United Kingdom were problematic (Citron & Ginsparg, 1995)]. This result is in line with recent studies which also reveal that the prevalence academic misconduct in developing countries is significantly higher than in highly developed countries (Pupovac et al., 2017; Xie et al., 2021). The empirical results showed remarkable variation across disciplines: while the incidence of incorrect borrowing is relatively low in physics, earth science, and humanities, scientists from social sciences, chemistry, and applied fields have engaged in plagiarism more regularly.

We proposed that socialization with a scientific ethos would be different for those who engaged in the international community in comparison to those who preferred to stay local. We measured the globalization of academic disciplines by calculating the share of publications indexed in the global citation database in the overall output of academic disciplines. In general, disciplines closely follow the pattern: a higher level of globalization is associated with a lower percentage of borrowed text (with chemistry as an exemption, which expresses an unexpected pattern of a globalized field with a high level of borrowed text). Even after running regression models with a number of controls, the effect is significant. We also found that that text borrowing is less prevalent in research-oriented institutions supporting global standards in their academic ethos.

We suggest that further research on structural factors explaining academic misconduct requires special efforts to improve the level of conceptualization of disciplinary variance. Horbach and Haifmann (2019) demonstrated that the extent of text-recycling is associated with differences in publication cultures among scientific disciplines. Seeber et al. (2019) considered the role of disciplinary differences in social structure to explain the incidence of self-citations as an opportunistic response to the use of metrics. However, less attention has been paid to the specifics of disciplinary conventions regarding dominant academic norms. Authors with plagiarized dissertations might not perceive themselves as scientists involved in the blatant disregard of norms as they might not prioritize the norm of complete originality or even have alternative norms. They could rationalize their actions, for example, by borrowing technical parts of the text (descriptions of experiments) or using the work of colleagues to which they have contributed (as when a supervisor writing a doctoral dissertation uses dissertations of candidates of sciences defended under their supervision).

Regarding the empirical prospect, it might be fruitful to examine whether academic disciplines express different patterns of incorrect text borrowing. Researchers usually use the percentage of borrowed text as the primary dependent variable without any attempts to categorize different types of plagiarism. We suggest that online software provides the opportunity to deepen our understanding of academic plagiarism if we focus on the practice of text borrowing. The relevant research questions include the following: Which parts of text are borrowed more often? Where are they located? How long are they? Do scientists copy substantial sections, or rather segments of small size? We suggest that the remarkable difference between disciplines might be evident regarding the different types of academic plagiarism. It is also possible that dominant rationalizations that scientists use to explain why their behaviors differ from socially accepted patterns are reflected in the dominant practice of text borrowing.

The prevalence of academic misconduct in developing countries is still an underdeveloped topic (Vrana, 2018; Zhang, 2010); although their output in global science is increasing, national policies on research integrity are still being created. Plagiarism in non-Western countries is usually considered in the context of authors’ attempts to publish something in international journals (Biagioli, 2012). Struggling with this challenge, they borrow different portions of articles to get through the standard of an Anglophone paper. However, we studied dissertations written in a local language published before the pressure to publish internationally. It seems that plagiarism in developing countries is related to more than the desire to cut corners and publish internationally at any cost. The general attitude to the norm of originality might be different, especially in disciplines (social sciences and applied fields) that do not follow the global publication culture. Local conventions to the originality of academic texts exist outside of opposition to the global norm. Studying academic misconduct in developing countries only through English-language publications might distract researchers’ attention from explanations why scientists may ignore the global norm of originality.

International publishing requires absorbing strict ethical standards regarding scholarly conventions of proper referencing. Authors and editors may be less familiar with traditional scholarly conventions in developing countries. Research shows that although global editors have expressed a mainstream view on ethical standards, the attitude of non-Anglophones is a little less rigorous than that of the Anglophones (Zhang & Zhang, 2016). The international experience that varies at the level of academic disciplines allows one to get familiar with standards and reproduce more mainstream views on academic research behavior. Even if we studied one national case, the same pattern might exist in other contexts, e.g., authors engaged in international publishing are more exposed to other ethical standards that result in changing local attitudes. However, the exact answer requires comparative data from other developing countries that might be a fruitful topic for further research.

The findings may be limited by relying on the similarity of checking software which has several inevitable drawbacks. First, the plagiarism rate might be underestimated due to false negatives—the cases of plagiarism not detected by the Antiplagiat software. The obvious source for false negatives is translational plagiarism, when an academic text published in English or other languages is translated into the local language and then used with minor changes without reference to the original source. Unfortunately, these cases are extremely difficult to detect, although different approaches to address them have been recently discussed (Memon, 2020). In this regard, our results might be biased towards revealing higher plagiarism rates for dissertations from less internationalized scientific disciplines in which authors rely primarily on Russian sources. In contrast, authors from more globalized disciplines might use translational plagiarism more often, but the software was not designed to detect it. Besides, authors might use various instruments to avoid the detection of similar texts, including synonym replacement or substituting letters from the Russian alphabet with similar letters from other alphabets. However, Antiplagiat specialists regularly monitor and counteract these techniques to inflate a paper's originality percentage.

## Footnotes

1 For more information, please review the Antiplagiat user guide: https://docs.antiplagiat.ru/en/html/private_user_guide.html.

2 The preliminary analysis of the sample of dissertations is available in Kopotev et al. (2021).

3 The algorithm was developed and applied by Antiplagiat’s programmers specifically for this study. We are grateful to Y. Chehovich, Executive Director of Antiplagiat, whose technical assistance made it possible to obtain data about text overlap with high precision.

4 Around 8% out of five thousand Russian journals in RICS are indexed in WoS. These journals are considered globalized because they can be reached through international circuits of knowledge distribution.

5 The results raise the question of how the considerable number of dissertations with the significant amount of plagiarized text were defended. By formal documents, the organizations with dissertation councils have been required to use antiplagiarism software since 2012, and until this moment, a dissertation might not have been checked at all. Even when the practice of obligatory checking was introduced, the councils might not have evaluated the results with proper attention; instead, they might have used the threshold (10 or 20%) to detect whether a dissertation contains plagiarized text. Using thresholds is widely spread among Russian academics despite the Antiplagiat’s instruction that this practice might lead to erroneous conclusions.
